# Research on Mine-Personnel Helmet Detection Based on Multi-Strategy-Improved YOLOv11

**DOI:** 10.3390/s25010170

**Published:** 2024-12-31

**Authors:** Lei Zhang, Zhipeng Sun, Hongjing Tao, Meng Wang, Weixun Yi

**Affiliations:** School of Coal Engineering, Shanxi Datong University, Datong 037000, China; dtblack84@cumt.edu.cn (L.Z.); 220857002141@sxdtdx.edu.cn (H.T.); 230857002119@sxdtdx.edu.cn (M.W.); 230857002147@sxdtdx.edu.cn (W.Y.)

**Keywords:** GCB-YOLOv11, mine personnel helmet detection, safety regulations, object detection

## Abstract

In the complex environment of fully mechanized mining faces, the current object detection algorithms face significant challenges in achieving optimal accuracy and real-time detection of mine personnel and safety helmets. This difficulty arises from factors such as uneven lighting conditions and equipment obstructions, which often lead to missed detections. Consequently, these limitations pose a considerable challenge to effective mine safety management. This article presents an enhanced algorithm based on YOLOv11n, referred to as GCB-YOLOv11. The proposed improvements are realized through three key aspects: Firstly, the traditional convolution is replaced with GSConv, which significantly enhances feature extraction capabilities while simultaneously reducing computational costs. Secondly, a novel C3K2_FE module was designed that integrates Faster_block and ECA attention mechanisms. This design aims to improve detection accuracy while also accelerating detection speed. Finally, the introduction of the Bi FPN mechanism in the Neck section optimizes the efficiency of multi-scale feature fusion and addresses issues related to feature loss and redundancy. The experimental results demonstrate that GCB-YOLOv11 exhibits strong performance on the dataset concerning mine personnel and safety helmets, achieving a mean average precision of 93.6%. Additionally, the frames per second reached 90.3 f·s^−1^, representing increases of 3.3% and 9.4%, respectively, compared to the baseline model. In addition, when compared to models such as YOLOv5s, YOLOv8s, YOLOv3 Tiny, Fast R-CNN, and RT-DETR, GCB-YOLOv11 demonstrates superior performance in both detection accuracy and model complexity. This highlights its advantages in mining environments and offers a viable technical solution for enhancing the safety of mine personnel.

## 1. Introduction

Coal is not only the cornerstone of China’s energy security strategy but also a vital resource that underpins the development of the national economy [1]. With the ongoing advancement of science and technology, utilizing these innovations to systematically address the safety challenges associated with coal production is a crucial method to ensuring China’s energy security [2]. Coal mine intelligence, as a core technology, has been driving the high-quality development of the coal industry by integrating artificial intelligence with other scientific and technological methods. This method encompasses various critical areas, including coal mine safety and production [3,4,5]. Mine personnel, as direct participants in coal mining activities, have their personal safety closely linked to both individual and family well-being. Furthermore, it serves as a crucial guarantee for the sustainable development of coal-mining enterprises. Currently, a majority of coal mines have implemented video surveillance systems in production areas to monitor personnel and ensure compliance with safety-helmet regulations. However, the complex environment within mines presents objective interference factors, such as inconsistent lighting and physical obstructions, which hinder effective supervision throughout the entire process. Therefore, the development of an efficient and intelligent algorithm for detecting mine personnel and safety-helmet objects, along with the implementation of safety warnings based on the detection outcomes, holds significant importance in enhancing the level of safety production within coal mines.

Traditional object-detection methods can be categorized into two primary components. The first component involves the manual extraction of features from the object image, while the second component entails transferring these features to a classifier for classification. Dalal et al. [6] employed the Histogram of Oriented Gradients (HOG) to extract features from mine personnel and utilized Support Vector Machines (SVMs) for object classification, subsequently achieving detection results. However, traditional object detection methods rely on manual operations across multiple steps, resulting in unsatisfactory overall detection performance. This limitation makes it challenging to meet detection requirements in complex backgrounds.

With the advancement of science and technology, deep learning has brought revolutionary changes to the fields of image recognition and object detection. As a significant branch of deep learning, Convolutional Neural Networks (CNNs) have demonstrated exceptional performance in object-detection tasks due to their powerful feature-extraction capabilities. Object-detection algorithms based on deep learning are primarily categorized into two-stage and single-stage networks. Two-stage networks, such as Region-Based Convolutional Neural Networks (R-CNNs) [7], depend on regional proposals. These algorithms first extract candidate regions from the image and subsequently classify and locate these regions [8]. In contrast, single-stage networks, including the Single-Shot MultiBox Detector (SSD) [9] and You Only Look Once (YOLO) [10], utilize bounding box regression. These algorithms combine image classification and prediction within a single network, significantly enhancing detection efficiency. Single-stage networks are widely utilized across various sectors due to their effective detection capabilities.

In recent years, an increasing number of scholars are concentrating on the detection and investigation of personnel safety helmets within related fields. Li et al. [11] introduced a multi-scale self-attention module within the feature extraction network of YOLOv3, which enhances the attention given to helmet features and increases the proportion of effective features, thereby achieving more accurate helmet detection. However, their model exhibits high complexity and is not suitable for mining scenarios that demand stringent real-time performance. Xie et al. [12] proposed an enhanced YOLOv4 helmet detection algorithm that incorporates the SE-Net module to improve the feature extraction capabilities of the backbone network. Additionally, it employs the DenseASPP module to mitigate feature transmission loss and optimizes the extraction of global contextual information, resulting in commendable performance in complex environments. However, the computational overhead associated with the DenseASPP module constrains the algorithm’s advantages in lightweight deployment and diminishes real-time detection efficacy. Yang et al. [13] employed MobileNetV3 for helmet feature extraction based on YOLOv5, aiming to reduce the number of parameters and computational demands of the network. They subsequently implemented DIoU-NMS to enhance the model’s ability to recognize occluded objects, thereby decreasing hardware costs while ensuring higher detection accuracy. However, in scenarios characterized by uneven illumination or complex occlusions within mining environments, there remains a need for further improvement in detection accuracy. Zhang et al. [14] proposed a DeDi-Transformer image-defogging algorithm aimed at enhancing the clarity of individuals and helmets in workface surveillance images. Subsequently, they introduced supervised zero-convolution in YOLOv9, which significantly improves helmet-recognition accuracy. However, this method necessitates additional preprocessing steps for mine video streams, thereby increasing the complexity of the system. Di et al. [15] proposed a downhole personnel-detection method based on an enhanced Transformer architecture, utilizing a lightweight Swin Transformer network in place of ResNet. This method not only reduces computational overhead but also enhances the model’s focus on personnel within images. Additionally, the attention module of the Swin Transformer has been reconfigured to achieve precise localization of individuals. However, it is noteworthy that the system operates at a real-time detection rate of 32 frames per second, and its adaptability for detecting personnel in dynamic environments remains inadequate. Shao et al. [16] introduced the ShuffNetV2 network to address the feature channel-isolation issue arising from the ELAN module in YOLOv7. They employed group convolution to enhance the computational foundation of the feature block, thereby improving the representation of mine-personnel features. Ultimately, this algorithm achieved a detection accuracy of 89.4% for mine personnel. However, the inclusion of its ACmix module within the neck network resulted in an increase in the overall parameters of the model, thus limiting sufficient computational capacity for underground equipment.

The aforementioned studies have enhanced the detection of personnel and helmets by incorporating mechanisms such as attention, lightweight networks, and multi-scale feature-extraction modules. However, due to the complexities inherent in the mining working face environment, existing detection methods remain susceptible to both false negatives and false positives under conditions characterized by uneven illumination and equipment obstruction. This limitation adversely impacts not only the accuracy of detection but also the reliability of safety warnings. For this paper, the main contributions are as follows:

(1). The standard convolution method is susceptible to generating redundant feature information during the extraction of features related to downhole personnel and safety helmets. This redundancy can result in issues such as an increased number of parameters and elevated floating-point computations. To address these challenges, we employed Group Shuffle Convolution (GSConv) as a replacement for the standard convolution within the backbone network. This method aims to optimize feature extraction efficiency, enhance detection accuracy, and reduce the computational cost of the model.
(2). As the depth of the network increases, the feature maps processed by C3K2 tend to contain a significant amount of irrelevant information, such as background elements. These extraneous data adversely impact both the accuracy and real-time detection capabilities of the model. To address this issue, we have redesigned the C3K2 module and introduced a novel C3K2_FE module. This new design enhances the model’s focus on key objects, specifically personnel and safety helmets, by integrating Faster_block and ECA attention mechanisms.
(3). To enhance the efficiency of feature fusion for two object types across varying scales, we implemented a bidirectional feature pyramid network (Bi FPN) mechanism at the neck [17]. This method improves multi-scale feature-fusion capabilities through skip connections, effectively addressing issues related to feature loss in complex environments and enhancing fusion efficiency under such conditions.

The remainder of this paper is structured as follows: Section 2 provides a comprehensive overview of the principles and methodologies employed in this study, including the key components of GCB-YOLOv11. Section 3 presents a thorough account of the experiments and analyses conducted, including the improvement experiment, the ablation experiment, and the comparative experiment. Section 4 discusses the limitations of the model and proposes future optimization strategies. Finally, Section 5 summarizes the accomplishments of this study.

## 2. Method and Principle

### 2.1. YOLOv11 Model

**Figure 1:**
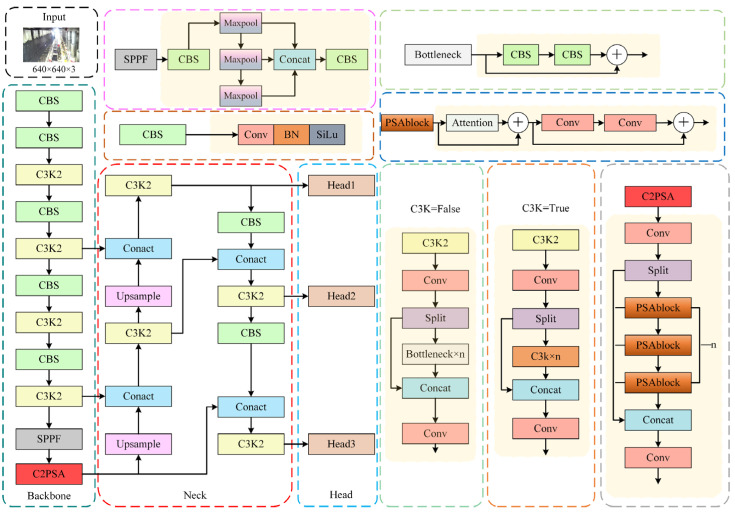
YOLOv11 model structure.

YOLO is a classical single-stage object-detection algorithm [18,19,20,21,22,23] and is one of the most significant representatives in the current field of object detection due to its advantages in speed, accuracy, interpretability, and wide applicability. The YOLOv11 model, as the latest development in the YOLO series, further enhances the performance and practicality of the algorithm. Moreover, YOLOv11 introduces several innovative features compared to previous models in the YOLO series. YOLOv11 architecture consists of four main components: input, backbone, neck, and head. Its structure is illustrated in Figure 1. The input component utilizes Mosaic [24] technology to enhance image quality. The backbone component incorporates the C3K2 module, a faster version of the Cross-Stage Partial (CSP) module, which is based on the original C3 module. The introduction of C3K2 enhances the model’s feature extraction capabilities. It integrates a C2PSA module, which functions similarly to the attention mechanism, positioned behind the Spatial Pyramid Pooling Fast (SPPF) layer to further improve feature extraction. The neck section utilizes the Feature Pyramid Network (FPN) and Path Aggregation Network (PAN) architectures. FPN is employed to construct feature pyramids, while PAN is used for feature aggregation. The head section performs object detection based on the features provided by the neck.

YOLOv11 achieved exceptional detection performance on the COCO dataset, demonstrating high-precision detection capabilities while maintaining lightweight characteristics in complex backgrounds. The YOLOv11 model can be categorized into five variants, YOLOv11n, YOLOv11s, YOLOv11m, YOLOv11L, and YOLOv11x, based on the network’s depth. This model is typically deployed on edge devices in comprehensive mining face scenarios, which require efficiency and lightweight design. Consequently, this paper selects YOLOv11n as the baseline model for researching the detection of mine personnel and helmet usage.

#### 2.1.1. C3 Module and C3K2 Module

**Figure 2:**
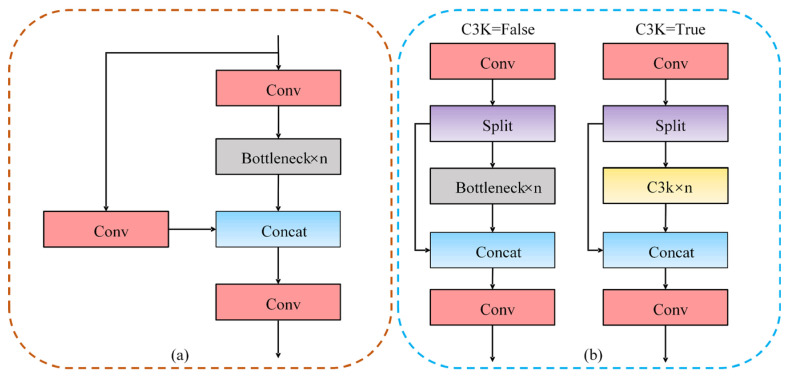
Two types of residual structures: (**a**) C3 structure and (**b**) C3K2 structure.

The C3 module is primarily designed based on the concept that CSPNet extracts the shunt mechanism and integrates it with the residual module, where the main branch gradient of CSP serves as the Bottleneck module. It employs the parameter n to control the number of stacked C3 modules, allowing the model to adapt to various network depths. The structure of C3 module is illustrated in Figure 2a, and it consists of three convolutional modules of size 1 × 1 and n residual modules. It incorporates two methods for processing the input-feature maps. The first method employs a two-branch method, where one branch utilizes two convolutional modules, while the other outputs the original feature maps; these two outputs are then combined. The second method omits the residual modules and directly outputs the feature maps following the operation of the convolutional modules.

The C3K2 module is designed with reference to the C2f module in YOLOv8 to enhance the extraction of object-feature information. Its structure is illustrated in Figure 2b. The C3K2 module selects the residual structure based on the value of the parameter C3K. If C3K is set to true, the C3K structure is employed; if it is set to false, the Bottleneck structure is utilized. Moreover, the parameter n governs the number of stacks of various structures. C3K2 is analogous to the two-branch method of the C3 module when processing the input-feature maps; however, there will be a discrepancy in the selection of the residual structure. The shallow network C3K is configured to false, while the deep network C3K is set to true.

#### 2.1.2. C2PSA Module

**Figure 3:**
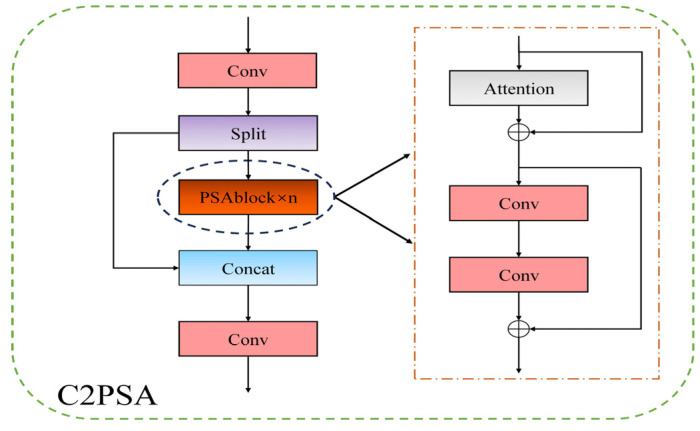
C2PSA module structure.

The C2PSA module incorporates the Pyramid Split Attention (PSA) mechanism, which optimizes the model’s efficacy in processing multi-scale features of the object based on the Squeeze-and-Excitation (SE) attention mechanism, thereby enhancing the model’s ability to extract object features. Furthermore, PSA module can be stacked multiple times, allowing the model to perform more effectively when handling multi-level features. The structure of C2PSA is illustrated in Figure 3. After a single convolutional operation on the input-feature map, the process branches into two paths. The left branch outputs the original-feature map, while the right branch undergoes operations through n PSA modules before being combined with the output from the left branch, resulting in the final feature map.

#### 2.1.3. Sort Detection Head

**Figure 4:**
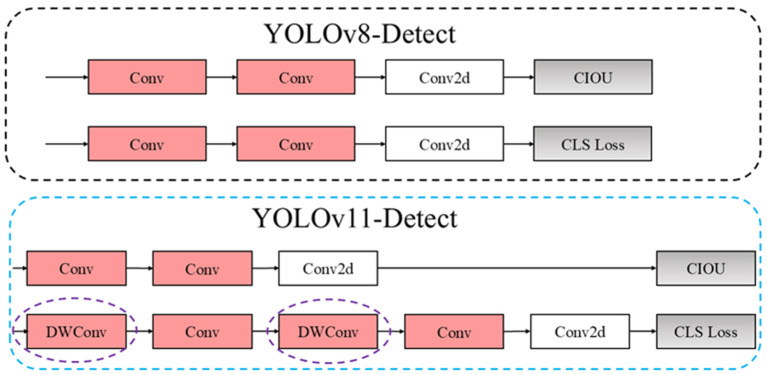
Comparison of YOLOv11 and YOLOv8 model detection head structures.

In the model detection head section, YOLOv11 continues to follow the design principles established in YOLOv8, which effectively ensures the stability of the model’s post-processing. The classification detection heads of YOLOv11 and YOLOv8 are illustrated in Figure 4. YOLOv11 incorporates two depth-separable convolutions, specifically depthwise convolution (DWConv), which is intended to reduce the number of parameters and computations required by the model, thereby lowering the computational cost. In this context, Conv2d denotes the 2D convolution, CIOU refers to the loss function, and CLS Loss indicates the object classification loss.

### 2.2. GCB-YOLOv11 Network Structure

**Figure 5:**
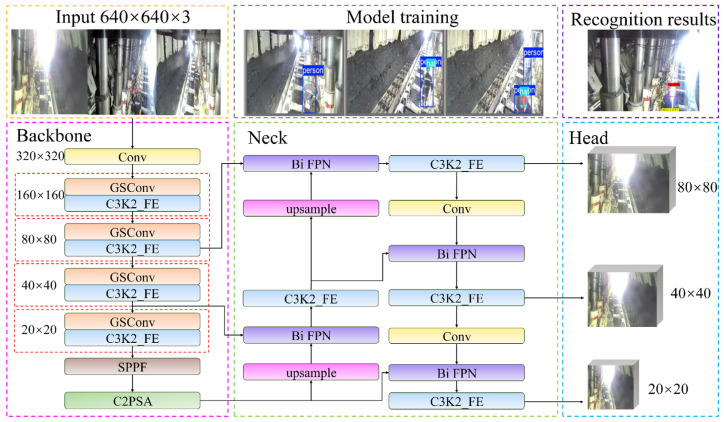
GCB-YOLOv11 network structure.

As illustrated in Figure 5, the overall architecture of GCB-YOLOv11 is primarily composed of four components: input, backbone, neck, and head. Within the backbone network, a standard convolution is initially employed to reduce the image dimensions to 320 × 320 pixels. Subsequently, four GSConv modules are utilized to replace the standard convolution for down-sampling operations, further reducing the image size to 20 × 20 pixels. In addition, the C3K2_FE module further enhances feature representation through the implementation of Faster-block and ECA attention mechanisms. The neck structure employs the Bi FPN mechanism, which effectively integrates feature maps of varying scales via bidirectional feature fusion, thereby improving the model’s capability to assimilate multi-scale objects. The neck structure employs the Bi FPN mechanism, which effectively integrates feature maps of varying scales via bidirectional feature fusion, thereby improving the model’s capability to assimilate multi-scale objects. After conducting feature extraction and fusion, the model produces feature maps of varying dimensions (80 × 80, 40 × 40, and 20 × 20) at the head layer. Each of these maps encompasses information regarding location, classification, and confidence levels.

#### 2.2.1. GSConv Module

**Figure 6:**
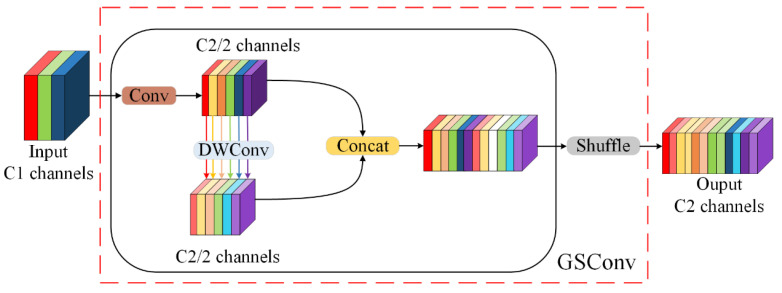
GSConv structure.

The YOLOv11 model utilizes standard convolution to extract feature information. Standard convolution (Conv) performs convolution operations across three channels simultaneously, with the number of convolution kernels matching the number of output channels. This can result in feature redundancy during the extraction of image features, thereby increasing the model’s complexity. In lightweight networks such as MobileNetV2 [25] and ShuffleNet [26], separable convolutions are commonly utilized to improve detection speed and reduce model complexity. GSConv adopts this design through a strategy module that integrates standard convolution (Conv), depthwise convolution (DWConv), and shuffling techniques. The core innovation of GSConv lies in the grouping and random channel shuffling of the input-feature maps. The structure of GSConv is illustrated in Figure 6. The feature map with an input-channel count of C1 first undergoes a standard convolution, which preserves the feature information while halving the input-channel count to produce an output-channel count of C2. Subsequently, the feature information from the Conv layer is fused with the output of the DWConv layer to maintain more channel connections and further reduce model complexity. Finally, the outputs of the Conv and DWConv layers are concatenated for channel reordering, which increases randomness and enhances feature representation.

In a mining environment, personnel and helmets may become partially obscured due to uneven lighting conditions or equipment occlusion. The GSConv method significantly enhances the model’s feature extraction capabilities through group convolution and channel blending operations. This method enables the capture of more subtle and diverse features associated with underground personnel and helmets, thereby improving detection accuracy. Secondly, the mine site typically has limited computational resources. GSConv addresses this challenge by reducing both computational complexity and the number of parameters through grouped convolution. This method allows the model to decrease its overall complexity while maintaining high performance, making it well-suited for real-time monitoring requirements.

#### 2.2.2. C3K2_FE Module

The backbone network of YOLOv11 consists of a series of convolutional layers and residual modules. During the feature-extraction process for detecting mine personnel and safety helmets, a significant amount of background information may be captured. As the network deepens, numerous feature maps can contain complex and irrelevant information, leading to a slower inference speed and challenges in meeting edge deployment requirements in mining environments. To address this issue, we have reconfigured the C3K2 module and proposed the C3K2_FE module, which incorporates the Faster_block [27] structure and the Efficient Channel Attention (ECA) [28] mechanism.

**Figure 7:**
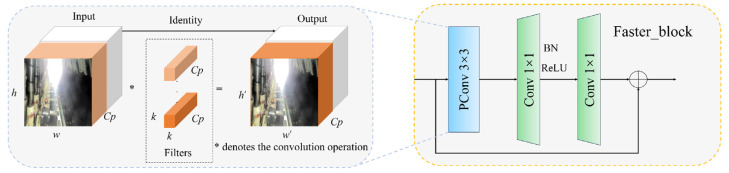
Faster_block structure.

Faster_block is proposed by Faster Neural Networks (FasterNet), with the core idea of reducing redundant computations and memory access to improve the network’s real-time performance while maintaining or enhancing detection accuracy. The structure of the Faster_block is illustrated in Figure 7., which primarily consists of one PConv module and two Conv modules. The PConv module deviates from the standard convolution operation by applying it only to a subset of the key channels, thereby reducing model complexity and enhancing real-time detection performance. Assuming the input-feature map has dimensions of *h* × *w* × *c*, the output feature map has dimensions of *h*′ × *w*′ × *c*′, and the convolution kernel has dimensions of *k* × *k*, where *Cp* denotes the quantity of partial convolution channel, we can express the complexities of Conv and PConv in terms of floating-point operations per second (FLOPs) as follows. F_Conv_ denotes the complexity associated with standard convolution, whereas F_PConv_ signifies the complexity related to PConv.

(1) $$F_{Conv}=h^{\prime }\times w^{\prime }\times c^{\prime }\times c\times k\times k$$

(2) $$F_{\mathrm{PConv}}=h^{\prime }\times w^{\prime }\times c^{\prime }\times k\times k$$

Assuming that PConv only operates on 1/4 of the input-feature channel, i.e., Cp/C = 1/4, according to Formulas (1) and (2), the FLOP of PConv is only 1/16 of Conv. Therefore, the design of Faster_block using PConv can reduce model complexity and accelerate model inference speed.

**Figure 8:**
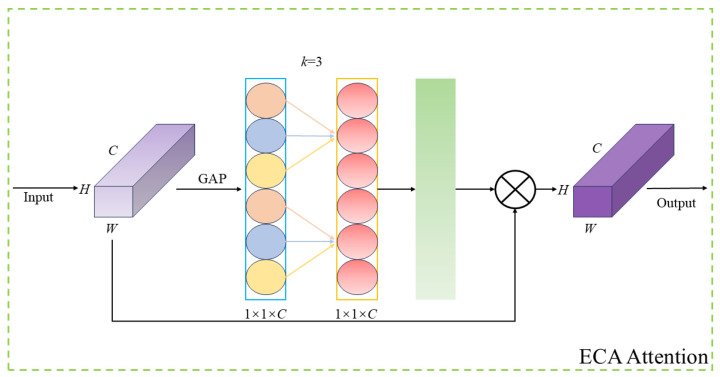
ECA attention mechanism structure.

To prevent the loss of model accuracy and to avoid issues such as insufficient feature extraction caused by the Faster_block module, an ECA attention mechanism was incorporated at the end of the C3K2 architecture. This addition aids the C3K2_FE module in more effectively acquiring contextual information, enhancing focus on key features, and ultimately improving detection accuracy. The ECA attention mechanism is capable of adaptively adjusting the weights of different channel features, allowing the C3K2_FE module to focus more effectively on significant features while suppressing irrelevant ones. The structure of ECA attention mechanism is illustrated in Figure 8. Initially, ECA attention mechanism performs a Global Average Pooling (GAP) operation on the feature map, followed by a one-dimensional convolution to facilitate cross-channel interaction and obtain the weights for each distinct channel. These weights are then multiplied by the corresponding sections of the original feature map to produce the final output feature map. In Figure 8, k represents the number of cross-channels, while C denotes the number of input channels.

**Figure 9:**
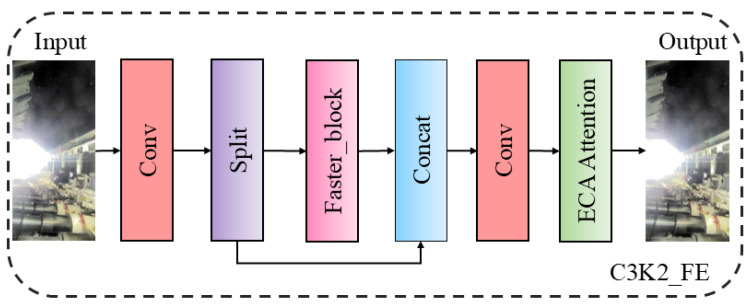
C3K2_faster structure.

In summary, C3K2_FE consists of six components: an input layer, two convolutional layers, a split layer, a Faster_block layer, an ECA attention mechanism, and an output layer. Its structure is illustrated in Figure 9. After passing through the convolutional layer, the feature map is bifurcated, and the convolved feature map re-enters the Faster_block layer for further feature extraction. Subsequently, the two branches perform a concatenation operation to achieve feature fusion, resulting in the final output after the application of the ECA attention mechanism.

The C3K2_FE module integrates the Faster_block and ECA attention mechanisms. The Faster_block enhances the system’s ability to operate stably at high frame rates while ensuring timely responses to safety events by accelerating model detection. This capability is particularly crucial for real-time monitoring systems in mining environments. In mining environments, where complex backgrounds and various occlusion scenarios may render object features less conspicuous, the ECA mechanism enables the model to concentrate on effective feature areas. This focus ultimately enhances detection accuracy.

#### 2.2.3. Bi FPN Mechanism

**Figure 10:**
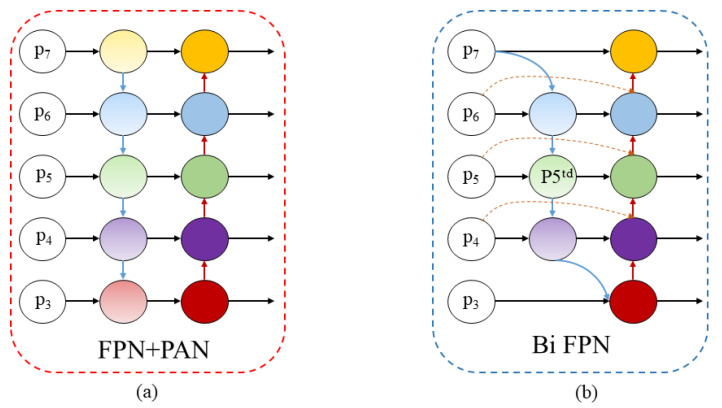
Feature pyramid structure: (**a**) FPN + PAN structure and (**b**) Bi FPN structure.

YOLOv11 integrates Feature Pyramid Networks (FPNs) and Path Aggregation Networks (PANs) at the feature fusion stage, utilizing a pyramid architecture to combine features of varying scales. The FPN + PAN structure is illustrated in Figure 10a, where the majority of the input to the PAN network is derived from the output of the FPN. This can result in the loss of certain features, particularly those related to the characteristics of mine personnel in different poses, which may lead to false positives in the model. Additionally, the FPN + PAN structure contains numerous nodes, which increases the computational cost of the training process. An object within the mining environment may manifest at various scales and locations. The Bi FPN effectively integrates feature information across these different scales, thereby enhancing the model’s capability to detect underground personnel and helmet features of varying sizes. The structure of Bi FPN is illustrated in Figure 10b. Compared to FPN + PAN, Bi FPN features bidirectional connections that transmit feature information through two pathways: top-down and bottom-up. This design retains more contextual information and improves feature-transmission efficiency. Additionally, Bi FPN removes edge nodes with inadequate fusion capabilities, streamlining the network architecture, reducing model training costs, and facilitating cross-fusion of nodes at various levels, thereby enhancing feature fusion across different scales. Finally, Bi FPN incorporates a learnable weight to discriminate the significance of different input features. The yellow dashed line in Figure 10b indicates the weighted fusion method implemented by Bi FPN through the addition of skip connections. Taking the P_5_ node as an example, the feature-fusion mechanism of the 5th level node is as follows, where *w*_i_ and *w*_j_ represent different weight parameters; *ε* = 0.0001; *I_i_* represents input features; *R* represents up-sampling or down-sampling; *Conv* denotes separable convolution; $P_{5}^{td}$ indicates the intermediate node of the P_5_ level; $P_{5}^{in}$ refers to the input feature of the fifth-level node; $P_{6}^{in}$ pertains to the input feature of the sixth-level node; $P_{5}^{out}$ signifies the output feature of the fifth-level node; and $P_{4}^{out}$ represents the output feature of the fourth-level node. Additionally, *ω*′ symbolizes the weight parameter.

(3) $$P=\sum_{i=0}\frac{\omega_{i}*I_{i}}{\varepsilon +\sum_{j=0}\omega_{j}}$$

(4) $$P_{5}^{\mathrm{td}}=Conv\left(\frac{\omega_{1}\cdot P_{5}^{in}+\omega_{2}\cdot R\left(P_{6}^{in}\right)}{\omega_{1}+\omega_{2}+\varepsilon }\right)$$

(5) $$P_{5}^{out}=Conv\left(\frac{\omega_{1}^{\prime }\cdot P_{5}^{in}+\omega_{2}^{\prime }\cdot P_{5}^{td}+\omega_{3}^{\prime }\cdot R\left(P_{4}^{out}\right)}{\omega_{1}^{\prime }+\omega_{2}^{\prime }+\omega_{3}^{\prime }+\varepsilon }\right)$$

## 3. Experiment and Analysis

### 3.1. Experimental Environment Configuration

The operating system used for this experiment is Windows 10. The deep-learning framework employed is PyTorch 2.2.0, and the Python version is 3.10. The GPU utilized is the NVIDIA GeForce RTX 3060 Ti (NVIDIA, Santa Clara, CA, USA), while the CPU is the Intel (R) Core (TM) i7-9700K (Intel, Santa Clara, CA, USA) CPU running at 3.60 GHz. The system is equipped with 32 GB of RAM, and CUDA 12.1.0 (NVIDIA, Santa Clara, CA, USA) is used for computational acceleration. The model training parameters are presented in Table 1.

### 3.2. Experimental Dataset

**Figure 11:**
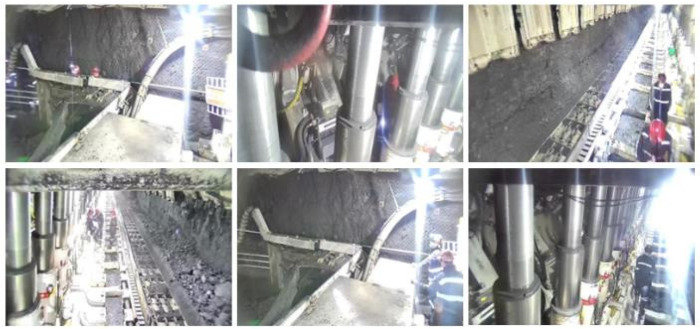
Sample example of dataset.

The dataset of mine personnel and safety helmets used in this experiment was obtained from video surveillance of the fully mechanized mining face in the coal mine. By extracting frames from the video footage and annotating them, a total of 5000 images of mine personnel and safety helmets were collected. Figure 11 presents a selection of the photos within the dataset. Upon completing the data collection, Labellmg (version number is 1.8.6)was utilized to annotate the images. The labels are classified into “person” and “hat”, where “hat” represents the safety helmet and “person” represents the mine personnel. The dataset was divided into training, validation, and testing sets in a ratio of 8:1:1.

### 3.3. Model Evaluation Indicators

In this experiment, mean average precision (mAP@0.5) is used as the evaluation metric for model accuracy. Parameters (Params) and FLOPS serve as the evaluation metrics for model complexity, while frames per second (FPS) is employed to assess detection speed. The calculation process for mAP@0.5 is as follows:

(6) $$P=\frac{\mathrm{TP}}{\mathrm{TP}+\mathrm{FP}}$$

(7) $$R=\frac{\mathrm{TP}}{\mathrm{TP}+\mathrm{FN}}$$

(8) $$\mathrm{AP}=\int_{0}^{1}P\left(R\right)\mathrm{dR}$$

(9) $$\mathrm{mAP}=\frac{\sum_{1}^{N}{\mathrm{AP}}_{i}}{N}$$

Among these terms, P denotes precision, R represents recall, TP indicates true-positive samples, FP indicates false-positive samples, FN indicates false-negative samples, and N represents the number of object categories. Average precision (AP) is defined as the area under the precision–recall curve and the coordinate axes, reflecting the model’s detection accuracy for a specific category. However, for object-detection tasks involving two or more categories, this indicator cannot serve as a unified measure. Mean average precision (mAP) is the average of average precision (AP) across all categories. mAP@0.5 represents the average accuracy of each category with an Intersection-over-Union (IoU) threshold of 0.5 between the predicted bounding box and the ground truth box, making it one of the most important metrics for evaluating model detection performance. Frames per second (FPS) indicates the model’s capacity to process a number of images per second; a higher FPS signifies faster detection speed and enhanced real-time performance. Additionally, a larger number of parameters (Params) and FLOPs in a model correlate with increased complexity, suggesting that more computational resources are required. The unit for Params is megabytes (M), while the unit for FLOPs is gigaflops (G).

### 3.4. Experimental Results

#### 3.4.1. Model Training Analysis

**Figure 12:**
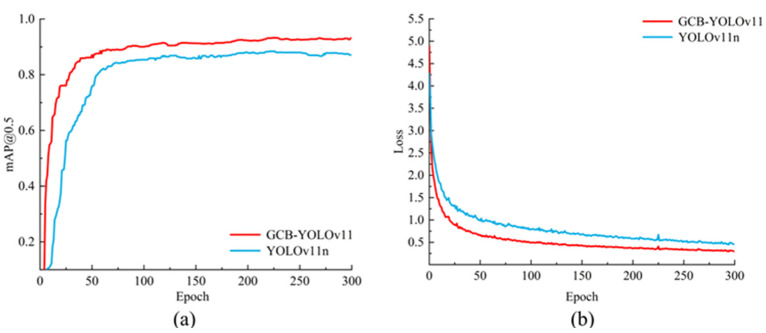
Comparison of training process curves between two types of models: (**a**) mAP@0.5 curve and (**b**) loss curve.

To evaluate the effectiveness of GCB-YOLOv11, we recorded its loss curve and mAP@0.5 curve to analyze the model’s training performance. Figure 12a illustrates the loss curves of two model types on the training set, with the horizontal axis representing the number of training iterations. As the number of training iterations increases, the training loss values for both models decrease, indicating that they can continuously and accurately learn the characteristics of mine personnel and safety helmets throughout the training process. Figure 12b displays the performance of the two model types in terms of the mAP@0.5 curve. Over 300 training rounds, the mAP@0.5 curve of GCB-YOLOv11 consistently remains higher than that of YOLOv11n. Around 220 training rounds, both curves begin to converge. The mAP@0.5 for GCB-YOLOv11 stabilizes at approximately 93.6%, while the mAP@0.5 curve for YOLOv11n stabilizes at around 90.3%.

**Figure 13:**
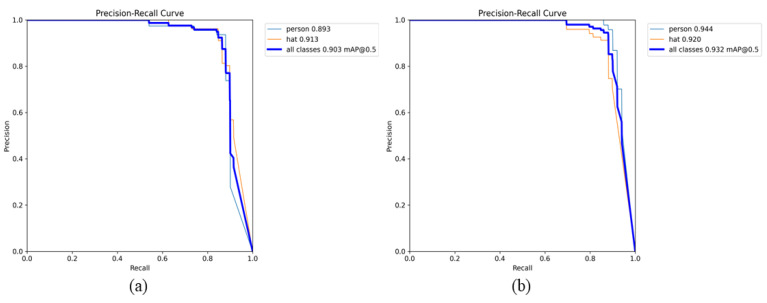
Comparison of P-R curves of two types of models on the validation set: (**a**) P-R curve of YOLOv11n and (**b**) P-R curve of GCB-YOLOv11.

To facilitate the evaluation of the average accuracy of the GCB-YOLOv11 model across two categories, we compared the precision–recall (P-R) curves of the YOLOv11n and GCB-YOLOv11 models using the validation set. The P-R curves for both models are illustrated in Figure 13. The area under the curve, in relation to the coordinate axes, indicates that the GCB-YOLOv11 model achieves high precision and recall rates. Its average accuracy for detecting mine personnel and safety helmets is 94.4% and 92.0%, respectively, representing an improvement of 5.1% and 0.7% over the YOLOv11n model. This suggests that the GCB-YOLOv11 model demonstrates superior detection accuracy for both mine personnel and safety helmets.

#### 3.4.2. Ablation Experiment

The core objective of the ablation experiment is to assess the impact of a specific factor on system performance or outcomes by systematically adding or removing it. To evaluate the optimization effect of incrementally incorporating each module into the baseline model, we designed an ablation experiment, and the results are presented in Table 2. The baseline model utilized in this study is YOLOv11n. The symbol “—” indicates that the mechanism has not been introduced, while the symbol “√” signifies that the mechanism has been introduced. Model A incorporates the GSConv module, while Model B integrates the C3K2_FE module. Lastly, Model C employs the Bi FPN module. After substituting the four standard convolutions in the baseline network with GSConv, we observed an improvement of 0.6% in mAP@0.5. Additionally, there was a reduction of 0.3 M in the number of parameters and a decrease of 0.7 G in floating-point computations. These results indicate that GSConv is capable of extracting more effective feature information while simultaneously reducing model complexity, thereby enhancing overall model performance. Using the C3K2_FE module in isolation, the mAP@0.5 increased by 0.8%. Additionally, there was a reduction of 0.4 M in the number of parameters and a decrease of 1.6 G in floating-point computations, while FPS improved by 6.3%. These results indicate that the C3K2_FE module effectively mitigates interfering factors within the feature maps, thereby enhancing detection accuracy. Furthermore, it is evident that this module significantly reduces model complexity. Introducing the Bi FPN mechanism in the neck component resulted in a 0.2% improvement in mAP@0.5. Additionally, there was a reduction of 0.7 M in parameter count and 0.2 G in floating-point computations, indicating that the Bi FPN mechanism effectively integrates feature information across different scales and enhances feature fusion efficiency. Model D is developed based on Model A and incorporates Model B. The mAP@0.5 has improved by 1.2%. Additionally, the parameter count and floating-point computations have decreased by 0.8 million and 2.1 gigaflops, respectively. Overall, the model is progressively aligning with the ideal direction. Model E represents the final enhanced version discussed in this article, which builds upon Model D by integrating features from Model C. This model demonstrates an improvement of 3.3% in mAP@0.5, while simultaneously reducing the parameter count and floating-point computations by 1.0 M and 2.0 G, respectively. Additionally, it achieves an increased frame rate of 90.3 frames per second. Through the aforementioned analysis, it is evident that the GSConv, C3K2_FE, and Bi FPN modules all play significant roles in enhancing model performance. Notably, among these components, C3K2_FE demonstrates the most substantial impact on improving both detection accuracy and real-time performance. It achieves a detection accuracy of 91.1% and a real-time processing rate of 87.7 frames per second, thereby facilitating further lightweight optimization of the model. The second component is GSConv, which enhances the model’s feature extraction capabilities while significantly reducing redundant information within the features. Finally, the Bi FPN mechanism is introduced, which offers a multi-scale feature fusion method that facilitates enhanced interaction between GSConv and C3K2_FE.

In summary, the GCB-YOLOv11 model introduced in this paper demonstrates superior accuracy and enhanced real-time performance when compared to the baseline model. Furthermore, it validates the feasibility of the optimization module proposed herein.

#### 3.4.3. Contrast Experiment

We selected the current State-of-the-Art (SOTA) models, which primarily include YOLOv3-Tiny, YOLOv5s, YOLOv8s, YOLOv11n, Fast R-CNN [29], RT-DETR [30], EfficientDet [16], HRHD-YOLOv8 [31], and BLP-YOLOv10 [32]. Among these models, Fast R-CNN and RT-DETR serve as representatives of larger architectures that typically exhibit high accuracy. However, their considerable model complexity leads to slower detection speeds, thus posing challenges in meeting the real-time monitoring and deployment requirements in mining operations. EfficientDet, in contrast, is a lightweight model characterized by low complexity and commendable real-time detection capabilities. However, it poses challenges in maintaining high levels of detection accuracy. The models YOLOv3-Tiny, YOLOv5s, YOLOv8s, YOLOv11n, HRHD-YOLOv8, and BLP-YOLOv10 exhibit a certain balance between detection accuracy and real-time performance. However, in complex environments, the feature-extraction capabilities of these models are inadequate for effectively capturing subtle object features. This limitation adversely impacts overall detection performance and results in frequent instances of missed detections. Therefore, to evaluate the detection performance of the GCB-YOLOv11 model for identifying mine personnel and safety helmets in underground environments, we conducted comparative experiments with the aforementioned models using the same dataset and experimental conditions. The specific results of these experiments are presented in Table 3. By analyzing the performance of these models based on mAP@0.5, we can draw several conclusions regarding the Params, FLOPs, and FPS metrics:

(1). In terms of detection accuracy, the model proposed in this article demonstrates a significant advantage, with a mAP@0.5 of 93.6%. This performance surpasses that of YOLOv3 Tiny, YOLOv5s, YOLOv8s, YOLOv11n, Fast R-CNN, RT-DETR, EfficientDet, HRHD-YOLOv8, and BLP-YOLOv10 by margins of 4.9%, 3.5%, 3.6%, 3.0%, 3.7%, 2.1%, 8.1%, and 4.3% respectively. These results indicate that the GCB-YOLOv11 model exhibits superior detection accuracy compared to other models and performs effectively in mining environments.
(2). Regarding model complexity, the GCB-YOLOv11 model proposed in this paper exhibits a parameter count of 1.6 million and a floating-point computation requirement of 4.5 billion. These metrics demonstrate superior performance compared to nine other classes of models, particularly when contrasted with the two larger categories: Fast R-CNN and RT-DETR. Furthermore, the relatively lower model complexity associated with Fast R-CNN suggests that the GCB-YOLOv11 model is well-suited for deployment in underground coal mine environments.
(3). In terms of real-time detection, the models YOLOv3-Tiny, YOLOv5s, YOLOv8s, EfficientDet, BLP-YOLOv10, and GCB-YOLOv11 demonstrate commendable performance. Notably, the FPS of GCB-YOLOv11 surpasses that of YOLOv3-Tiny, YOLOv5s, and YOLOv11n. Although models such as Fast R-CNN and RT-DETR exhibit slightly lower FPS compared to YOLOv8s, they still achieve an impressive FPS of 90.3 f·s^−1^, thereby satisfying the requirements for real-time processing.

In summary, the GCB-YOLOv11 model demonstrates exceptional detection performance, characterized by a commendable balance between detection accuracy and real-time efficiency. When compared to other SOTA models, it not only achieves high levels of detection accuracy and speed but also maintains low parameter counts and minimal floating-point computations.

#### 3.4.4. Visualization Experiment

**Figure 14:**
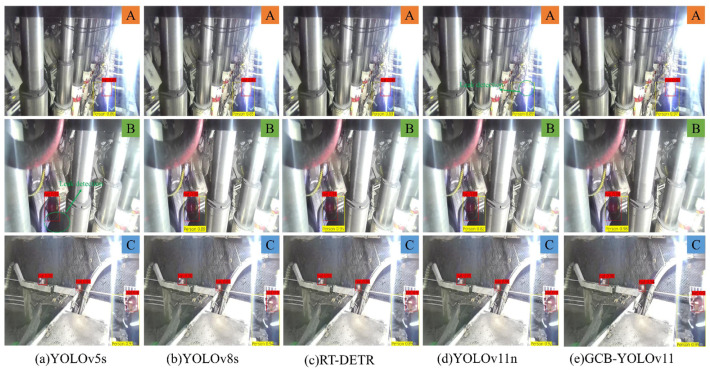
Different model detection results.

To more intuitively illustrate the detection capabilities of the GCB-YOLOv11 model concerning mine personnel and safety helmets, the author selected three precision models for comparison: YOLOv5s, YOLOv8s, and RT-DETR. The results are depicted in Figure 14, where A represents a reflective scene, B represents an occluded scene, and C represents a multi-object scene. In Scenario A, YOLOv11n failed to detect safety helmets, while the other three models accurately identified both mine personnel and safety helmets. In Scenario B, YOLOv5s failed to detect any partially occluded mine personnel, while YOLOv11n and YOLOv8s did not achieve a confidence level of 90% for detecting mine personnel. In contrast, RT-DETR and GCB-YOLOv11 demonstrated excellent detection performance. In Scenario C, all five models accurately identified mine personnel and safety helmets, with GCB-YOLOv11 exhibiting a significantly higher confidence level compared to the other detection models. By comparing the detection performance of various models, it is evident that GCB-YOLOv11 effectively mitigated the missed detection issues observed with YOLOv5s and YOLOv11n. Compared to YOLOv8s and RT-DETR, GCB-YOLOv11 not only possesses higher confidence but also demonstrates the best overall model performance.

**Figure 15:**
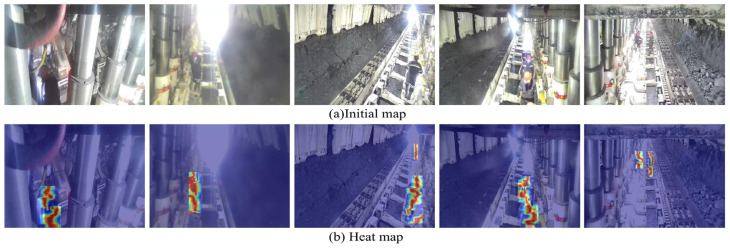
GCB-YOLOv11 heatmap.

This article utilizes Gradient-Weighted Class Activation Mapping (Grad-CAM) [33] to generate a heatmap for the GCB-YOLOv11 model, aiming to analyze the model’s focus on various features. As illustrated in Figure 15, the heatmap is categorized into red, yellow, and blue, reflecting the order of contribution. The red regions predominantly encompass areas where mine personnel and safety helmets are present, indicating that GCB-YOLOv11 model places greater emphasis on these feature areas.

## 4. Discussion

The GCB-YOLOv11 model has demonstrated exceptional detection performance and effectiveness in identifying mine personnel and safety helmets in fully mechanized mining environments. However, certain limitations and new research avenues must be considered in future work. These include the following:

GCB-YOLOv11 demonstrates consistent performance in fully mechanized mining environments. However, in settings such as excavation faces, the model may exhibit weak adaptability to new data, which can adversely affect its detection performance due to various influencing factors. Future research should focus on integrating a broader range of data concerning personnel and safety helmets from diverse mining scenarios to further improve the model’s generalization capabilities.

(1). This study adopts YOLOv11 as the benchmark model for enhancement. Additionally, there are several high-performance deep-learning networks that can be utilized to develop models for detecting underground personnel and safety helmets. Further research is necessary to explore the integration and analysis of various methods in the future.
(2). The real-time detection performance of GCB-YOLOv11 could potentially be further improved by incorporating lightweight network concepts into the model. However, finding the right balance between detection accuracy and real-time performance is a critical consideration for future research.
(3). Although the Bi FPN mechanism has been introduced to enhance multi-scale feature fusion, GCB-YOLOv11 may still experience issues related to leakage or false detection when identifying very small or highly overlapping objects. This is primarily due to the small size and dense distribution of personnel and helmets in the mining environment.
(4). The environment at the mine working face is complex and dynamic, often characterized by disruptive factors such as dust and smoke. While GCB-YOLOv11 demonstrates commendable performance on the current dataset, its detection efficacy may be compromised in various mining contexts or under extreme environmental conditions. Therefore, further validation and optimization are essential to enhance its adaptability to diverse environments.

In conclusion, while GCB-YOLOv11 has achieved notable success in detecting mine personnel and safety helmets, it is essential to continuously explore new strategies to address its potential shortcomings and further improve the performance of the object detection model in practical engineering applications.

## 5. Conclusions

(1). The GCB-YOLOv11 model, designed for detecting underground personnel and safety helmets in coal mines, is proposed. This model integrates three significant modules, GSConv, C3K2-FE, and Bi FPN, which are based on the baseline model. GSConv allows the model to group and shuffle features, thereby reducing complexity while maintaining detection accuracy. The C3K2_FE module, developed from the C3K2 structure, is applied throughout the entire network. This module not only improves the model’s real-time performance but also achieves high detection accuracy. Additionally, the Bi FPN mechanism effectively enhances the fusion efficiency of features at different scales through cross-layer operations.
(2). Based on my self-constructed dataset of mine personnel and safety helmets, the GCB-YOLOv11 model was evaluated. The experimental results demonstrated that the average accuracy of the GCB-YOLOv11 model reached 93.6%, while its real-time detection performance achieved 90.3 f·s⁻¹. Additionally, the occurrence of missed detections was significantly reduced, effectively fulfilling the detection requirements for mine personnel and safety helmets. This provides a technical solution to enhance the safety of coal mine workers.

## Figures and Tables

**Table 1:** Model training parameters.

Parameter	Set Value
Image size	640 × 640
Training frequency	300
Batch	32
Training optimizer	SGD
Lr0	0.01
Weight attenuation coefficient	0.0005

**Table 2:** Ablation experiment.

Model	GSConv	C3K2_FE	Bi FPN	mAP@0.5/%	Params/M	FLOPs/G	FPS/f·s^−1^
Baseline	—	—	—	90.3	2.6	6.5	82.5
A	√	—	—	90.9	2.3	5.8	79.2
B	—	√	—	91.1	2.1	4.9	87.7
C	—	—	√	90.5	1.9	6.3	75.2
D	√	√	—	91.5	1.8	4.4	82.6
E(our)	√	√	√	93.6	1.6	4.5	90.3

**Table 3:** Comparison of performance indicators of different models.

Model	mAP@0.5/%	Params/M	FLOPs/G	FPS/f·s^−1^
YOLOv3-Tiny	88.7	9.5	14.3	83.3
YOLOv5s	90.1	5.4	13.9	82.0
YOLOv8s	90.0	9.8	23.3	90.4
YOLOv11n	90.3	2.6	6.5	82.5
Fast R-CNN	89.9	40.0	207.1	54.2
RT-DETR	91.5	42.8	130.5	59.7
EfficientDet	90.0	6.8	6.1	88.6
HRHD-YOLOv8	90.2	12.5	27.4	68.3
BLP-YOLOv10	89.6	3.28	9.8	80.0
GCB-YOLOv11	93.6	1.6	4.5	90.3

## Data Availability

All data generated or analyzed during this study are included in this published article. The original contributions presented in the study are included in the article, further inquiries can be directed to the corresponding author.
